# Systematic analysis of alternative first exons in plant genomes

**DOI:** 10.1186/1471-2229-7-55

**Published:** 2007-10-17

**Authors:** Wei-Hua Chen, Guanting Lv, Congying Lv, Changqing Zeng, Songnian Hu

**Affiliations:** 1 Key Laboratory of Genome Science and Information, Beijing Institute of Genomics, Chinese Academy of Sciences, Beijing, China; 2 Graduate School of Chinese Academy of Sciences, Beijing, China; 3 Nanyang Institute of Technology, Henan, China; 4 Bioinformatics, Heinrich-Heine-University, Duesseldorf, Germany

## Abstract

**Background:**

Alternative splicing (AS) contributes significantly to protein diversity, by selectively using different combinations of exons of the same gene under certain circumstances. One particular type of AS is the use of alternative first exons (AFEs), which can have consequences far beyond the fine-tuning of protein functions. For example, AFEs may change the N-termini of proteins and thereby direct them to different cellular compartments. When alternative first exons are distant, they are usually associated with alternative promoters, thereby conferring an extra level of gene expression regulation. However, only few studies have examined the patterns of AFEs, and these analyses were mainly focused on mammalian genomes. Recent studies have shown that AFEs exist in the rice genome, and are regulated in a tissue-specific manner. Our current understanding of AFEs in plants is still limited, including important issues such as their regulation, contribution to protein diversity, and evolutionary conservation.

**Results:**

We systematically identified 1,378 and 645 AFE-containing clusters in rice and *Arabidopsis*, respectively. From our data sets, we identified two types of AFEs according to their genomic organisation. In genes with type I AFEs, the first exons are mutually exclusive, while most of the downstream exons are shared among alternative transcripts. Conversely, in genes with type II AFEs, the first exon of one gene structure is an internal exon of an alternative gene structure. The functionality analysis indicated about half and ~19% of the AFEs in *Arabidopsis *and rice could alter N-terminal protein sequences, and ~5% of the functional alteration in type II AFEs involved protein domain addition/deletion in both genomes. Expression analysis indicated that 20~66% of rice AFE clusters were tissue- and/or development- specifically transcribed, which is consistent with previous observations; however, a much smaller percentage of *Arabidopsis *AFEs was regulated in this manner, which suggests different regulation mechanisms of AFEs between rice and *Arabidopsis*. Statistical analysis of some features of AFE clusters, such as splice-site strength and secondary structure formation further revealed differences between these two species. Orthologous search of AFE-containing gene pairs detected only 19 gene pairs conserved between rice and *Arabidopsis*, accounting only for a few percent of AFE-containing clusters.

**Conclusion:**

Our analysis of AFE-containing genes in rice and *Arabidopsis *indicates that AFEs have multiple functions, from regulating gene expression to generating protein diversity. Comparisons of AFE clusters revealed different features in the two plant species, which indicates that AFEs may have evolved independently after the separation of rice (a model monocot) and *Arabidopsis *(a model dicot).

## Background

Alternative splicing (AS) is an important mechanism, which contributes greatly to protein diversity by selectively using different sets of exons of one gene in different tissues or cells under certain circumstances [1-3]. It has been shown to exist in nearly all metazoan organisms, and was estimated to involve 30–70% of human genes [4,5]. However, AS variants identified so far are biased towards alternative exons that include coding sequences (CDSs) [6]. Actually, many AS isoforms use alternative first exons (AFEs) to regulate their expression and generate protein diversity. An AFE is the first exon of one splice isoform of a gene, but either located downstream of a corresponding AFE of other isoforms generated by the same gene, or absent from other isoforms altogether. It has been reported that this phenomenon also contributes to the complexity of gene expression [6,7].

To date, studies of AFEs have been focused mainly on mammalian genomes, especially mouse and human. It has been reported that of the full-length genes in the RIKEN databases, about 9% contained AFEs in mouse [8] and more than 18% contained AFEs in human [9]. AFEs could be produced by alternative promoter usage. Some AFEs merely change the 5'-untranslated region (5'-UTR) to exert regulation on translational efficiency or the efficiency or destination of the transcripts' transportation out of the nucleus. In this case, the shared downstream exons contain the translation start codons (ATGs), and thus have the same open reading frames (ORFs) and produce identical proteins [6,10-12]. In other cases, AFEs contain alternative transcription start sites (ATGs), which could result in protein variants that differ in the N-termini [2,13,14] or in novel proteins [15,16].

Up until now, only few studies have analyzed AFEs in plants. For example, SYN1 in *Arabidopsis *was shown to produce two isoforms with distinct alternative first exons [17]. Recently, a large-scale study of AFEs in rice has discovered 46 potential AFE-containing clusters, and has shown their involvement in tissue-specific transcription [14]. But our knowledge about AFEs in plants is still limited. Here, we used a systematic approach to analyze their contribution to protein diversity and their evolutionary conservation between rice (a model monocot) and *Arabidopsis thaliana *(a model dicot).

## Methods

### Systematic detection of AFEs in plant genomes

To compile our AFE data sets, we downloaded the following data sets of rice (*Oryza sativa *L. *ssp. Japonica*) and *Arabidopsis *from public databases: full-length cDNAs, expressed sequence tags (ESTs), reference sequences (NCBI refseq) and mRNAs (Table 1). Genome location and exact gene structure were determined for each of the cDNA sequences using the GMAP program [18]. We excluded sequences that showed low similarities with the genome sequence (<95% identities and <90% coverage for reference genes and full-length cDNAs; <90% identities and <90% coverage for ESTs), did not map onto a unique genomic region, or were derived from organelles (mitochondrion and chloroplast). All information was loaded into MySQL databases for further analysis.

**Table 1:** Acquired data

Species	Sequence	Datasets	Database
*Oryza sativa *L. *ssp. Japonica*	General EST	1,211,078	NCBI dbEST
	mRNA	23,309	NCBI CoreNucleotide
	Full-length cDNA	32,127	KOME**
	Genome		IRGSP* Release 4.0
*Arabidopsis thaliana*	General EST	734,275	NCBI dbEST
	mRNA	30,476	NCBI CoreNucleotide
	Full-length cDNA	15,294	RIKEN RAFL***
	Genome		NCBI Genomes

*IRGSP stands for International Rice Genome Sequencing Project

**KOME stands for Knowledge-based Oryza Molecular biological Encyclopedia

*** RAFL stands for RIKEN *Arabidopsis *Full-length cDNA clones

We first grouped full-length cDNAs and reference genes into clusters on the genome if they mapped onto the same genomic region, were orientated on the same strand, and had overlapping sequences. Within each cluster, members were further grouped according to their gene structures. ESTs were then added into the existing clusters. An EST was either added as a member of an existing gene structure, or as a new gene structure in a cluster according to the location of the first exon on the genome. ESTs that could not be grouped into a unique gene structure in one cluster were discarded. After adding ESTs, we counted the number of ESTs for each gene structure in each cluster. To produce reliable results, we discarded gene structures that consisted of only one EST.

Since only full-length cDNAs in our data sets could guarantee the reliability of transcription start sites (TSSs) and the first exons, we searched for AFEs in clusters that contained full-length cDNAs and had at least two distinct gene structures. We defined the first exon of a cluster as the 5'-most of all first exons among gene structures that contained full-length cDNAs. Then other gene structures in the same cluster were compared with this first exon to identify possible AFEs.

Within each AFE-containing gene cluster, we determined major and minor types of alternative first exons by calculating numbers of their supporting ESTs. A first exon type was marked as 'major' type if it had more supporting ESTs than any other first exon in the cluster; else it was marked as 'minor'.

### Statistical analysis of AFEs

Based on the alignment positions of AFEs, we determined the chromosomal distribution of AFE clusters in rice and *Arabidopsis*.

To identify possible factors that govern splicing sites selection in AFEs, such as splicing site strength, common motifs around splicing junctions, and secondary RNA structure formation around the splicing site, we performed the following statistical analyses of AFEs in rice and *Arabidopsis*. First, we examined splicing site quality of alternatively spliced first exons. By using exon annotations from GMAP, we extracted a 500-basepair window centered on each donor (5') splice site with sufficient flanking sequence, and used these data as input sequences to GeneSplicer [19] for splice site prediction.

Second, we analyzed whether AFEs tend to form secondary structures around splicing sites, which might potentially block the proper recognition of splice site signals and might thereby result in the skipping of the corresponding exon/intron. We used the program RNAfold of the Vienna RNA package [20] to predict folding for a 100-basepair window centered on each splicing site. The minimal folding energy (MFE, also known as optimal folding energy, OFE) was calculated for each input sequence. A lower MFE score indicates that the input sequence is more likely to form secondary structures.

Third, we used MEME [21] to search for possible common motifs shared by all or a subset of alternatively spliced exons and neighboring intron sequences.

### Annotation and functional classification of AFE-containing clusters

To annotate AFE-containing clusters, we compared either the reference gene or the longest full-length cDNA (if there was no reference sequence available) in each cluster with protein sequences in the Uniprot database [18] using BLAST-based tools. GO (Gene Ontology) terms were assigned according to Uniprot2GO associations downloaded from the website of the GeneOntology Consortium [22]. GO annotations were plotted using a web-based tool, WEGO [23]. Statistical significance of each GO category that was enriched or depleted among AFE-containing clusters was evaluated by calculating the hypergeometric distribution using the following equation:

p=f(x|M,K,n)=(Kx)(M−Kn−x)(Mn)
 MathType@MTEF@5@5@+=feaafiart1ev1aaatCvAUfKttLearuWrP9MDH5MBPbIqV92AaeXatLxBI9gBaebbnrfifHhDYfgasaacH8akY=wiFfYdH8Gipec8Eeeu0xXdbba9frFj0=OqFfea0dXdd9vqai=hGuQ8kuc9pgc9s8qqaq=dirpe0xb9q8qiLsFr0=vr0=vr0dc8meaabaqaciaacaGaaeqabaqabeGadaaakeaacqWGWbaCcqGH9aqpcqWGMbGzcqGGOaakcqWG4baEcqGG8baFcqWGnbqtcqGGSaalcqWGlbWscqGGSaalcqWGUbGBcqGGPaqkcqGH9aqpdaWcaaqaamaabmaaeaqabeaacqWGlbWsaeaacqWG4baEaaGaayjkaiaawMcaamaabmaaeaqabeaacqWGnbqtcqGHsislcqWGlbWsaeaacqWGUbGBcqGHsislcqWG4baEaaGaayjkaiaawMcaaaqaamaabmaaeaqabeaacqWGnbqtaeaacqWGUbGBaaGaayjkaiaawMcaaaaaaaa@4C62@

Where *M *= total genes classified by GO in an organism, *K *= number of genes classified by a specific GO category, *n *= total number of AFE-containing clusters classified by GO, *x *= number of AFE-containing clusters classified by a specific GO category, and *p *= probability that a GO category is significantly enriched or depleted.

### Tissue-specific expression of AFEs in rice and *Arabidopsis*

For the reliable detection of the tissue specificity of certain AFE isoforms, we adopted a strategy proposed by Qiang Xu *et al*. [5], namely 'tissue specificity scoring'. To this end, tissue specificity was measured by a tissue specificity score *TS *and two robustness values *r*TS and *r*TS~ (for details see Ref. [5]). High confidence (HC) tissue specificity was defined as *TS*>50, *r*TS>0.9 and *r*TS~>0.9, and low confidence (LC) was defined as *TS*>0, *r*TS>0.5 and *r*TS~>0.5.

### Cross-genome comparison of AFEs-containing orthologous genes

Orthologous relationship between rice and *Arabidopsis *were identified by using Inparanoid [24] with default parameter settings and with the Bootstrap option enabled. The output was parsed using a PERL script. Only genes that produced Bootstrap score = 100% were considered as orthologous.

### Functionality of AFE-containing clusters

We used the tool *GetORF *in the EMBOSS software package [25] to find putative open reading frames for every AFE-containing cluster. To assess the potential of AFEs to produce protein diversity, we divided the AFE-containing structures into three groups: i) AFEs in a certain cluster were not involved in the ORF and the downstream exons resulted in the same ORF for all AFEs; ii) AFEs contained alternative transcription start sites (ATG), but the downstream exons were identical; iii) AFEs contained alternative transcription start sites and the downstream exons were not identical.

In order to check if an AFE-containing structure generated transcripts containing premature stop codons (PTC) and could thus be degraded by nonsense-mediated decay mechanisms (NMD), the distance between the stop codon and the last 3' exon-exon junction was calculated. The NMD candidate was defined according to the 50 nt rule, as previously suggested [26]: If the measured distance was >50 nt, the AFE-containing structure was regarded as an NMD candidate.

## Results and discussion

### Systematic identification of AFEs in plant genomes

Based on comparisons of sequences from a large set of public databases, we identified 23,500 and 12,964 full-length-cDNA containing gene clusters in rice and *Arabidopsis*, respectively. These gene clusters represented about 42% (out of 55,890 gene loci from the TIGR Rice Genome Annotation Release 4) and 48.5% (out of 26,751 protein coding genes from the TAIR *Arabidopsis *Genome Annotation Release 6) of the total expressed genes in rice and *Arabidopsis*, respectively. From this data, we identified 1,378 and 645 AFE-containing clusters in rice and *Arabidopsis *clusters, respectively. In rice, ~5.9% of the expressed genes displayed AFE events. Compared with a recent estimate of ~4% based on 5'-end ESTs [14], which were obtained from CAP-technology-based cDNA libraries, our AFE ratio is slightly higher. This increase may result from i) our much larger collection of full-length cDNAs and general 5'-end ESTs, and/or ii) our potentially more sensitive detection method. In *Arabidopsis*, we observed a similar ratio (~5%) of expressed genes that contained AFE events.

**Figure 1:**
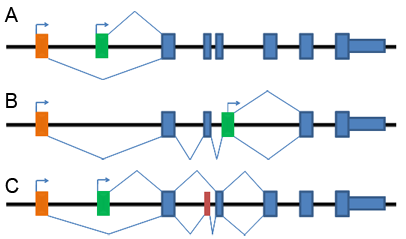
**Diagrammatic view of different types of AFE events**. Alternative first exons are highlighted in orange and green. Constitutive exons are drawn in dark blue. Other alternatively spliced exons are drawn in brown. (A). Type I AFE clusters. Alternative first exons are mutually exclusive in different gene structures. (B). Type II AFE clusters. The first exon of one transcript is (part of) a downstream exon of other transcripts. (C). Some AFEs are coupled with downstream alternative splicing events.

Based on the genomic positions of the first exons in a cluster, two patterns of AFEs were observed. Type I AFEs included those where the first exons were mutually exclusive and where most of the downstream exons were identical between gene structures within the same cluster (Figure 1A); Type II AFEs included those where the first exon of gene structure A existed as an internal exon of gene structure B (Figure 1B). It should be noted that sometimes a cluster could contain more than one type of AFEs.

From our data sets, Type II was the most abundant type of AFEs. Type II accounted for 90% (1,241 out of 1,378) of all the AFE events in rice, and 83% (546 out of 645) in *Arabidopsis *(Table 2). The average distance between the start sites of alternative first exons was 1,644 bp in *Arabidopsis*, and 1,141 bp in rice. Using the >500 bp interval proposed by Kouichi Kimura *et al*. [6] as a criterion, we estimated that at least 257 and 352 of the Type II AFE evens in rice and *Arabidopsis*, respectively, resulted from alternative use of different core promoters. By applying the same criterion to type I AFE events, we identified an additional 62 and 22 putative alternative promoter (PAP)-derived gene structures in rice and *Arabidopsis*, respectively. Although we could not determine the exact transcription start sites (TSSs) for non-full-length cDNA containing gene structures, our data suggested that the derived putative TSSs probably reflected true TSSs *in vivo*, as gene structures in each AFE cluster were supported by multiple general 5'-end ESTs from multiple cDNA libraries. Thus, we estimate that about ~23% and ~58% of AFE-containing gene structures were derived from alternative promoters in rice and *Arabidopsis*, respectively.

**Table 2:** Results of AFE analysis in rice and *Arabidopsis*

		Rice	Arabidopsis
Type I AFE		137	99
	N-terminal diversification	53	20
	Overlapping with functional domain	5	1
	Putative alternative promoter	62	22
	Both N-terminal and PAP	3	7
	NMD	47	10

Type II AFE		1,241	546
	N-terminal diversification	213	298
	Overlapping with functional domain	56	71
	Putative alternative promoter	257	352
	Both N-terminal and PAP	189	244
	NMD	237	42

Total		1,378	645

### Statistical characterization of AFEs in plant genomes

**Figure 2:**
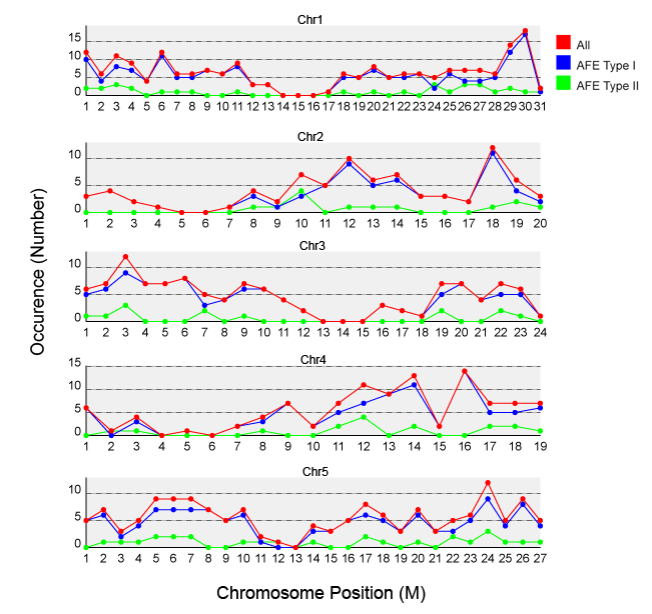
**Chromosomal distribution of AFE-containing clusters**. The distribution of AFEs on *Arabidopsis *chromosomes was determined using the alignment positions of AFE-clusters.

As shown in Figure 2, we detected no significant bias in the chromosomal distribution of AFEs in *Arabidopsis*. We also compared the distribution with relative gene density from the TAIR genome annotation, and did not detect any significant regional enrichment or depletion within chromosomes. A similar trend was also observed in the rice genome (see Additional File 1).

It is well documented that splice site strength plays important roles in splice-site selection and alternative splicing in mammalian genomes. Sequence composition around splice sites and its base pairing with the small nuclear RNA U1 regulate the inclusion rate of corresponding exons. To study whether similar mechanisms apply to plant genomes, we analyzed the 5' splice site (5'ss) strength of AFEs and compared it with that of constitutively spliced exons. As shown in Table 3, the results indicate that the 5'ss of type I AFEs is relatively weak compared to constitutive exons, in both rice and *Arabidopsis*. However, when taking the exon inclusion rate into account, we found significant differences between the two genomes. In *Arabidopsis*, the 5'ss strength of the major expressed AFE isoforms showed no statistical difference compared with that of constitutive exons (T-Test with *p *< 0.01), while the minor AFE isoform differed significantly from the constitutive exon in splice site strength (*p *= 3.2361e-012, Table 3). Conversely, in rice we observed similar 5'ss strengths between major and minor AFE isoforms. The analysis of type II AFEs revealed similar differences between rice and *Arabidopsis*: the 5'ss strength in both major and minor type II AFE isoforms of *Arabidopsis *was similar to that of constitutive exons, while the 5'ss strength of major AFE isoforms of rice was much lower compared to minor isoforms. These results suggest that different mechanisms are likely involved in the regulation of splicing-site selection or recognition in rice and *Arabidopsis*.

**Table 3:** 5' splice site analysis of AFEs

	Constitutive (± SD) *	AFE Type I			AFE Type II		
		
		Total	Major**	Minor**	Total	Major**	Minor**
Rice	9.310 ± 3.72	7.87 ± 4.11	7.75 ± 4.23	7.75 ± 3.91	8.61 ± 4.01	7.75 ± 4.03	8.98 ± 3.20
Comparison with constitutive sites ***		1.3063e-011	5.7841e-007	1.3907e-006	3.1057e-010	1.0233e-029	0.9846
Arabidopsis	8.00 ± 2.89	7.39 ± 3.23	8.20 ± 3.03	5.89 ± 3.07	8.44 ± 2.93	8.42 ± 2.84	8.40 ± 3.02
Comparison with constitutive sites ***		0.0013	0.4077	3.2361e-012	9.4224e-005	0.0062	0.0151

* The 5' splice site scores were predicted by GeneSplicer. Higher score indicates stronger splicing signal.

** Major and minor types of alternative first exons within each gene cluster were determined as described in the Methods section.

*** P-values were determined using *t-*tests.

We further investigated the tendency to form secondary structures of sequences surrounding the 5'ss of AFEs, as such structures were previously suggested to be able to regulate splice site recognition and splicing. We measured minimal folding energy (MFE) for a 100-base window centred on each 5'ss for AFEs as well as constitutive exons. As shown in table 4, the results indicated that AFEs of *Arabidopsis *were less likely to form secondary structures at the 5'ss compared to constitutive first exons, while AFEs in rice were significantly more likely to form secondary structures.

**Table 4:** secondary structure formation analysis at 5' splice sites of AFEs

	Constitutive (± SD) *	AFE Type I			AFE Type II		
		
		Total	Major**	Minor**	Total	Major**	Minor**
Rice	-19.22 ± 5.59	-23.61 ± 8.62	-24.28 ± 8.37	-23.00 ± 8.79	-22.45 ± 7.8	-24.7 ± 8.51	-20.37 ± 6.46
Comparison with constitutive sites ***		3.2796e-071	1.8749e-061	9.6957e-035	9.6069e-082	1.7511e-160	3.0208e-012
Arabidopsis	-17.80 ± 4.33	-15.09 ± 5.10	-14.59 ± 5.38	-15.60 ± 4.62	-16.52 ± 4.98	-16.47 ± 4.89	-16.46 ± 5.29
Comparison with constitutive sites ***		1.6711e-028	4.5892e-022	1.3987e-011	4.7938e-015	1.9863e-009	2.9444e-009

* Secondary structure formation was measured as Minimal Folding Energy (MFE) by MRNAFOLD. Lower scores indicate a higher likelihood of an input sequence to form a secondary structure;

** Major and minor types of alternative first exons within each gene cluster were determined as described in the Methods section.

*** P-values were determined using *t-*tests.

To investigate possible sequence motifs that might regulate the alternative use of first exons, we searched the sequences of AFEs and surrounding introns using the MEME program. Using a cutoff of 1E-5 for sequence alignments, we did not detect significantly enriched motifs in all or subsets of AFEs and surrounding sequences. This result indicates that either some regulatory sequences were too degenerative to be detected using MEME, or AFEs are regulated by other mechanisms than specific sequence motifs.

### Effects of AFEs on protein diversity and functional modulation

To study the biological implications of the alternative use of first exons, we examined whether the N-terminal coding regions were altered in AFEs. The N-terminals were considered to be altered when the putative Methionine start codon was located on the alternative first exons of both AFE types.

In type I AFE clusters (mutually exclusive first exons), the most common scenario involved AFE events that produced transcripts with identical ORFs. In these cases, a common downstream exon which contained the translation start site was shared by all gene structures in the cluster. From our data sets, 84 and 79 of AFE clusters in rice and *Arabidopsis*, respectively, were of this type. Because the protein structure remained unchanged, alterations between tissue or stage specificity were likely to be the main consequences in these cases.

In type II AFE-containing gene clusters, EST-only gene structures and full-length-containing ones often differed from each other by not only the alternative first exons, but also some downstream exons. Therefore, it was possible that the extra sequences in EST-only structures contained putative translational start codons, and consequently produced multiple protein variants. In our data, 213 and 298 type II AFE clusters in rice and *Arabidopsis *were of such cases, respectively. Most of these alternative start codons led to additional fragments at the N-termini of proteins. However, we identified some rare cases (five in rice and three in *Arabidopsis*, respectively) where AFEs resulted in multiple reading frames and thereby produced novel proteins.

In total, we identified 266 possible N-terminal changes in rice and 318 in *Arabidopsis *AFE-containing gene clusters. As shown in Table 2, a strong correlation existed between N-terminal protein changes and the use of putative alternative promoters in type II AFE clusters (as tested using Fisher's Exact Test, *p *< 0.01). It seemed that the distance between gene structures in a cluster contributed significantly to the N-terminal protein changes. Only a small proportion of type I AFE clusters generated protein diversity. The major contributor was the start codon location. We observed no connection between the 5'-end distance of the gene structures and alternative start codons.

We also investigated the effects of protein N-terminal changes on known functional protein motifs by comparing putative ORF translations of transcript isoforms with the NCBI Conserved Domain Database (CDD) [27]. As shown in Table 2, about 5~10% of N-terminal changes in type I AFE clusters overlapped with know functional protein domains in at least one of the isoforms, while 20~30% of N-terminal changes in type II AFE clusters did so. We found that ~5% of the functional alterations in type II AFE clusters involved whole domain additions and/or deletions. Such AFE-introduced protein modulation has the potential to result in complex functional regulation.

We noticed that, at least in some cases, the use of alternative first exons was coupled with downstream alternative splicing events (Figure 1C), which probably caused reading frame shifts and rendered the subsequent isoforms possible candidates for nonsense-mediated mRNA decay (NMD). We thus deduced the putative transcription isoforms for gene structures that did not contain full-length/reference sequences based on the approach from TAP [28]. We used the definition of premature termination codons (PTCs) as in-frame stop codons residing >50 bp upstream of the last 3' exon-exon junction, as previously reported [26]. Screening results indicated that about 284 and 52 of AFE transcription isoforms in rice and *Arabidopsis *produced NMD candidates, respectively. These frequencies were much smaller than those observed in the total of plant AS isoforms [26]. This discrepancy might partly result from the fact that AFE-coupled alternative splicing events are only a small subset of the total AS events in plants; it suggests that most of the AFE-containing events are functional, which is consistent with our analysis of the relationship between AFEs and protein diversity.

### GO classification of AFE-containing events

To investigate which kinds of genes were likely to use alternative first exons and what biological consequences AFEs could bring about, we first categorized AFE-containing clusters in rice and *Arabidopsis *according to the Gene Ontology classification. Then we used the whole genome GO categories from rice and *Arabidopsis *as references to calculate the probability that a GO category in the AFE-containing clusters was significantly enriched or depleted. As listed in Tables 5 and 6, although categories of diverse functions were observed, genes participating in enzymatic reactions and cellular processes were significantly enriched in both plants. Enrichment of AFE-containing clusters was also found for the functional categories of cellular process regulation, transporter, ATP binding, cell communication, and response to endogenous stimulus in rice. These results indicate that the complex transcription regulation mediated by AFEs might be indispensable for the adaptation to dynamic changes in the external and internal environments of plant cells. It appears plausible that when the environment changes, protein functions are fine-tuned by the addition and/or deletion of functional motifs at the N-termini, or protein localizations are re-assigned by altering signal peptides or transporter activities.

**Table 5:** Functional categories (GO) significantly biased in AFE-containing clusters in *Arabidopsis*

	GO category	AFE containing cluster	*P*-value*
Enriched**	cellular physiological process	327	0
	metabolism	297	0
	nucleotide binding	65	0
	catalytic activity	27	1.52E-10
	transferase activity	104	1.35E-09
	ligase activity	25	1.73E-08
	hydrolase activity	89	1.20E-07
	ubiquitin ligase complex	13	1.24E-07
	intracellular part	259	1.94E-07
	intracellular	265	2.42E-07
	cell part	368	7.82E-06
	membrane part	37	4.80E-05
	nucleic acid binding	91	0.000128
	lyase activity	18	0.000265
	localization	51	0.000476

Depleted	triplet codon-amino acid adaptor activity	0	5.61E-06

* *P*-value was calculated by the hypergeometric distribution. The cutoff is 1E-5.

** "Enriched" categories refer to those containing significantly more genes (observed) than expected. "Depleted" categories refer to those containing significantly less genes (observed) than expected.

**Table 6:** Functional categories (GO) significantly biased in AFE-containing clusters in Rice.

Enriched	GO category	AFE containing cluster	*P*-value
	metabolism	468	0
	cellular physiological process	595	0
	nucleotide binding	155	0
	hydrolase activity	144	0
	transferase activity	131	0
	oxidoreductase activity	79	0
	ion binding	65	0
	nucleic acid binding	147	1.02E-14
	helicase activity	17	2.78E-09
	catalytic activity	45	1.04E-08
	lyase activity	24	1.95E-08
	regulation of cellular process	50	3.95E-08
	regulation of physiological process	50	4.25E-08
	non-membrane-bound organelle	35	4.98E-08
	ligase activity	32	6.29E-08
	ATPase activity, coupled to movement of substances	20	7.01E-08
	organelle part	35	7.38E-08
	intracellular organelle part	35	7.38E-08
	membrane	208	1.32E-07
	carrier activity	27	2.15E-07
	membrane part	32	1.24E-06
	protein binding	26	1.66E-06
	ion transporter activity	23	2.67E-06
	ribonucleoprotein complex	23	1.38E-05
	microtubule associated complex	7	2.78E-05
	cell communication	22	3.91E-05
	amine binding	6	4.49E-05
	protein transporter activity	9	0.000192
	response to endogenous stimulus	13	0.000197
	unlocalized protein complex	5	0.000212
	cofactor binding	6	0.000212
	ATP-binding cassette (ABC) transporter complex	7	0.000245
	ubiquitin ligase complex	18	0.000306
	nuclear pore	3	0.000338

Depleted	membrane-bound organelle	860	1.47E-52
	intracellular organelle	878	9.04E-47
	intracellular part	905	4.36E-39
	intracellular	911	7.83E-38
	cell part	1,004	2.46E-33

**Figure 3:**
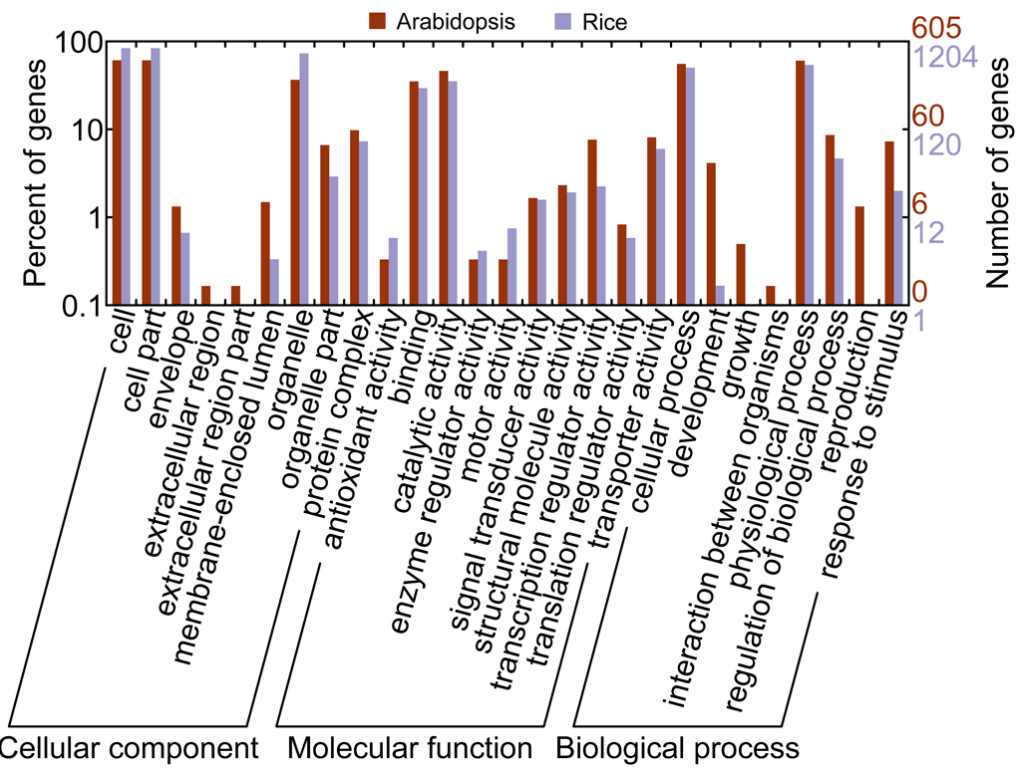
**Gene Ontology (GO) categories of AFE-containing clusters in rice and *Arabidopsis***. The genes were functionally categorized according to the Gene Ontology Consortium and level two of the assignment results were plotted here. 87% (1,204 of a total 1,378) AFE-containing clusters from rice and 94% (605 of a total 645) AFE clusters from *Arabidopsis *were classified by GO.

Several GO categories showed inconsistency between rice and *Arabidopsis *(Figure 3). For example, "intracellular part", "intracellular" and "cell part" were enriched in *Arabidopsis*, but were reduced in rice. Further studies are needed to elucidate such discrepancies.

**Figure 4:**
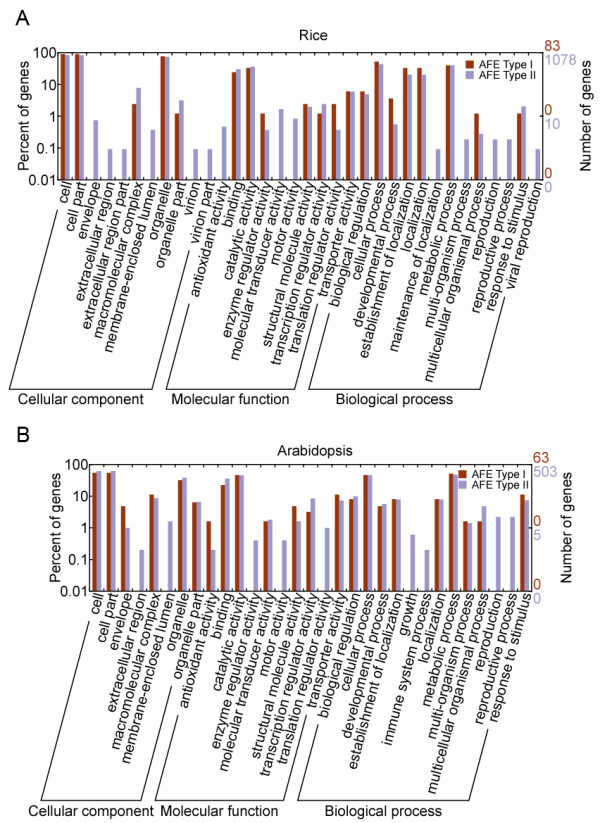
**Gene Ontology (GO) categories of two types of AFE-containing clusters in rice and *Arabidopsis***. The genes were functionally categorized according to the Gene Ontology Consortium and level two of the assignment results were plotted here. GO categories of two types of AFE-containing clusters were plotted for rice (A) and *Arabidopsis *(B), respectively.

We also compared functional differences between the two types of AFEs in rice and *Arabidopsis*. As shown in Figure 4, although there were differences in categories that contained only a few genes, such as "envelope", "molecular transducer activity" and "reproduction", none of these was statistically significant (Fisher's Exact Test *p *< 0.05). Thus, we concluded that there were no significant functional biases between type I and type II AFE clusters in rice and *Arabidopsis*.

One should note that at least one disadvantage of using GO classification is that GO mappings of identical gene products from different databases are sometime different, and so the results should be used with a certain degree of caution.

### Tissue- and development stage- specific expression of AFE isoforms in plant genomes

We adopted a method suggested by Qiang Xu *et al*. [5] to evaluate whether AFEs were involved in tissue- and/or developmental stage-specific expression. Tissue and developmental stage information were downloaded from the NCBI Library Browser classification. For those libraries with ambiguous or incomplete information in the Unigene database, we checked their dbEST entries and classified them accordingly. Then we calculated three scores for each AFE-containing gene, namely a tissue specificity score *TS *and two robustness values *r*TS and *r*TS~. As shown in Table 7, by using High Confidence criteria (HC, see Methods), we identified 390 and 31 AFE clusters involved in tissue-specific expression, as well as 273 and 44 AFE clusters involved in development-stage-specific expression, in rice and *Arabidopsis*, respectively. With slightly less stringent criteria (Low Confidence, LC, see Methods), the numbers of specifically expressed genes increased two to three-fold.

**Table 7:** Tissue- and development stage- specific expression of AFEs in rice and *Arabidopsis*

		Tissue specific*	Development stage specific*	Both
Rice	HC**	390	273	200
	LC**	914	713	624
Arabidopsis	HC	31	44	21
	LC	55	113	39

* Tissue- and development stage- specific gene expression were determined using the methods suggested by Qiang Xu *et al*.

** High confidence (HC) tissue specificity was defined as *TS*>50, *r*TS>0.9 and *r*TS~>0.9, low confidence (LC) was defined as *TS*>0, *r*TS>0.5 and *r*TS~>0.5 (see Methods)

In total, we estimated that around 20~66% of rice AFE clusters were regulated in an either tissue- or development-specific transcription manner. Our results are consistent with a previous report that AFEs are involved in tissue-specific transcription in rice [14]. Conversely, in *Arabidopsis*, we found only 5~18% of AFE-containing clusters to be expressed specifically in certain tissues and/or developmental stages.

### Evolutionary conservation of AFEs in plant genomes

To study the conservation of AFE events between rice and *Arabidopsis*, we used the longest reference gene or full-length cDNA in each AFE cluster as representative sequence. Ortholog relationships were identified by applying Inparanoid [24] to these sequences. To our surprises, only 19 AFE-containing gene pairs from rice and *Arabidopsis *were classified as orthologous groups, which accounted for only 1.4% of all AFE-containing gene clusters in rice and 2.9% in *Arabidopsis*. As shown in Figure 3, GO categories of AFE-containing gene clusters showed no biases between rice and *Arabidopsis *(Fisher's Exact Test, *p *< 0.05), indicating that evolutionary conservation exists in functional categories instead of individual genes in plant genomes.

## Conclusion

Based on our large scale general 5'-EST and full length cDNA alignments to the genomes of rice and *Arabidopsis*, we estimated that at least ~5% of expressed geneclusters in plants use alternative first exons. We further analyzed statistical features of these alternatively spliced exons and compared them with that of constitutively spliced exons. The results indicated that there could be more differences between AFEs from rice and *Arabidopsis *than generally anticipated. Expression analysis revealed that 20~66% of rice AFE clusters were regulated in either tissue- or development- specific manner, which was consistent with a previous report [14]. However, only 5~18% of *Arabidopsis *AFE clusters were involved in tissue- or development- specific expression. Although the GO classification of the AFE-containing clusters showed no functional biases between rice and *Arabidopsis*, only 19 groups of orthologous AFE-containing clusters were identified between the two plants. Considering that monocot and dicot plants may use different splicing machineries which are not completely compatible [29,30], we suggest that AFE events may have evolved independently after the separation of dicot and monocot lineages.

Although some of the AFE events were removed by nonsense-mediated mRNA decay (NMD), which constitutes an mRNA surveillance system, we found that the proportion of NMD coupled AFE events was much lower than that of the total set of alternative splicing evens in plants. Therefore AFE events appear particularly likely to create biologically functional transcription isoforms. Unlike a previous report [14], we have shown that the 49% and 19% of AFE events from *Arabidopsis *and rice affected the N-terminal protein sequences, and approximately 23% of rice and 57% of *Arabidopsis *AFE events may derive from the alternative use of multiple promoters. We anticipate that further studies of the relationship between AFEs and protein diversity in vivo will greatly enrich our knowledge about the complexity of gene expression regulation.

All analysis tools, database dumps and detailed description of methods are available upon requests, correspondence should be addressed to HuSN.

## Competing interests

The author(s) declares that there are no competing interests.

## Authors' contributions

SNH and WHC conceived the study. LvCY and CQZ collected the data and performed the statistical analysis. LvGT and WHC controlled and analyzed the data, and drafted the manuscript. All authors read and approved the final manuscript.

## Supplementary Material

Additional file 1 **Chromosomal distribution of AFE-containing clusters in rice genome**. The distribution of AFEs on rice chromosomes was determined using the alignment positions of AFE-clusters. Click here for file
