# Supplementary figures and images for: Systematic analysis of alternative first exons in plant genomes
Source: BMC Plant Biol. 2007 Oct 17;7:55. doi: 10.1186/1471-2229-7-55 (PMC2174465; doi:10.1186/1471-2229-7-55)

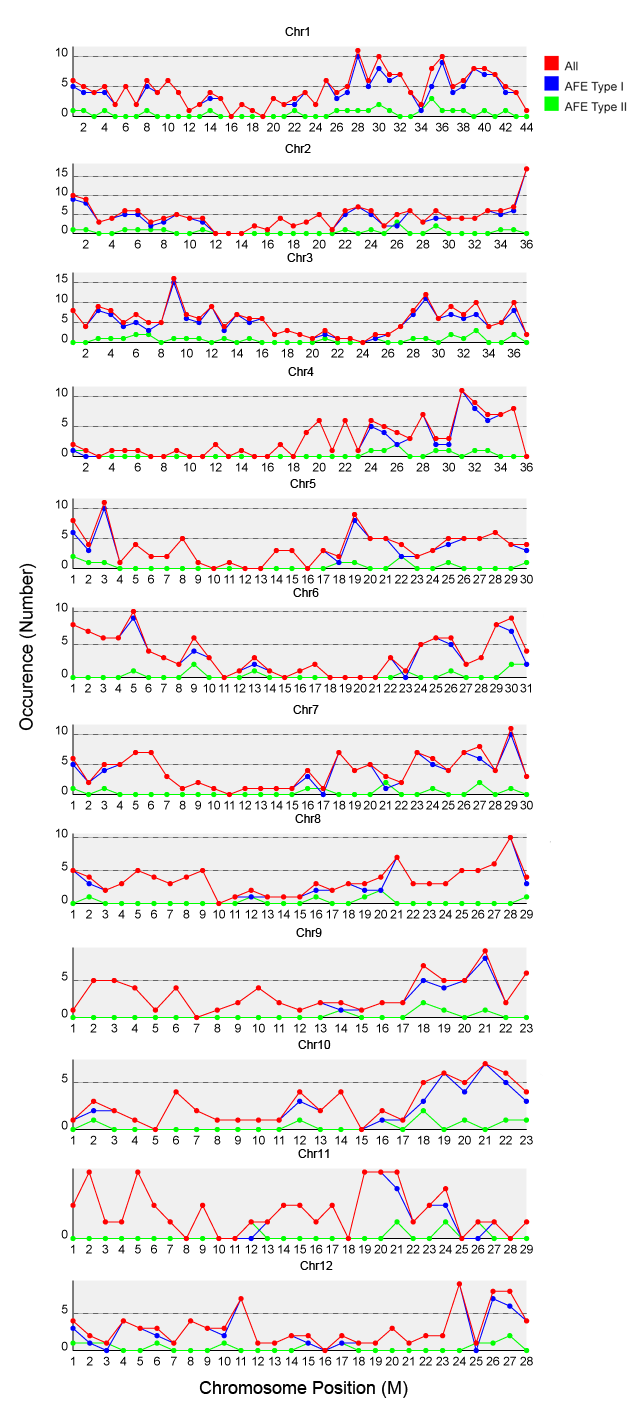

Supplement: Additional file 1 — Chromosomal distribution of AFE-containing clusters in rice genome. The distribution of AFEs on rice chromosomes was determined using the alignment positions of AFE-clusters. [file 1471-2229-7-55-S1.png]
